# BAY 61-3606, CDKi, and sodium butyrate treatments alter gene expression in human vestibular schwannomas and cause cell death *in vitro*

**DOI:** 10.3332/ecancer.2012.285

**Published:** 2012-12-20

**Authors:** Rohan Mitra, Indira Devi Bhagavatula, Rajalakshmi Gope

**Affiliations:** 1 Department of Human Genetics, NIMHANS, 2900 Hosur road, Bangalore 560029, India; 2 Department of Neurosurgery, NIMHANS, 2900 Hosur Road, Bangalore 560029, India

**Keywords:** Na-Bu, CDKi, BAY61-3606, human VS tumours, cell cycle proteins

## Abstract

**Background:**

Disrupted kinase and signaling pathways are found in many human cancers and they are implicated in carcinogenesis. Therefore, kinases have been important targets for the development of cancer therapeutics. Human vestibular schwannomas (VS) are the third most common intracranial tumours which occur in the vestibular branch of VIII^th ^ cranial nerve. Sodium butyrate (Na-Bu) is a potent histone deacetylase inhibitor (HDACi) and with therapeutic efficacy. Spleen tyrosine kinase (Syk) has been implicated in many immunological consequences and is a putative target for cancer treatment.

**Aims and objectives:**

The present study was undertaken in order to evaluate the effect Na-Bu, 2,4-Diamino-5-oxo-pyrimidine hydrochloride (CDKi), a broad spectrum kinase inhibitor and BAY 61-3606 (Syk inhibitor) on the survival of VS tumour tissues *in vitro * and their possible effects on cell survival/death and levels of a few key proteins in the treated cells as compared to the untreated cells.

**Materials and methods:**

Fresh tumour tissues were collected randomly from 16 patients with sporadic, VS tumours, minced into pieces and maintained in primary cultures. Twenty four hours later these cells were exposed to Na-Bu, BAY 61-3606 or CDKi. Forty eight hours after exposure, the tissue lysates were analysed by western blotting for expression of pRb and other proteins involved in cell survival/death.

**Summary and significance of the findings:**

The tissue samples used were positive for S100A protein, the maker for schwann cells confirming the VS tumour samples. The three individual treatments led to morphological change, DNA fragmentation and cell death and significantly reduced level of total and phosphorylated forms of pRb protein and drastically reduced EGF-R protein. These treatments also modulated levels of other proteins involved in cell survival/death such as PI3K, Caspase 3, TGF-β1, JNK, ASK1, Shh, NF-κB, p21^cip1/waf1^. The Untreated cells had uncleaved PARP-1 protein and the treated cells had cleaved PARP-1. The results show that the observed cell death in treated cells perhaps is mediated by modulation of the levels and processing of certain key proteins. The possible development of these components as therapeutics is discussed.

## Introduction

Human vestibular schwannomas (VS) arise from the vestibular branch of the 8th cranial nerve and are benign in nature. These tumours occur in both sporadic and familial forms. The sporadic VS tumours are generally unilateral, and the familial forms are bilateral and are also known as neurofibromatosis type 2 (NF2). Both the alleles of the NF2 gene are inactivated in all VS tumours and as a result these tumours lack the corresponding gene product, Merlin, or schwannomin. In familial cases, the NF2 tumours are known to occur at early ages as compared to sporadic cases. The symptoms associated with VS tumours include hearing loss, nausea, vertigo, tinnitus and facial paralysis, and hydrocephalus is found in severe cases. Due to its anatomical location, patients suffer great morbidity. Mortality rates are low due to this tumour; however, a higher rate of mortality is associated with secondary malignant tumours including glioma, meningiomas, etc. [1, 2].

The retinoblastoma gene (RB1) is the first tumour suppressor gene to be identified and it codes for a protein product, pRb, which has an important role in cell cycle during the G_0_-G_1_-S phase transition [3–5]. In addition, it has an important role in cell differentiation, apoptosis, neurogenesis, maintenance of telomere length, etc. [6–8]. pRb has 16 serine/threonine sites that are phosphorylated by the cyclin-CDK (cyclin dependant kinases) complexes, cyclin D/CDK4, cyclin E/CDK2 and cyclin A/CDK2, when there is mitogenic signal available in extracellular milieu [3–5]. The cyclin/CDK-mediated signalling mechanism and its effect on cellular proliferation are widely studied [9]. The spleen tyrosine kinase (Syk) is known to interfere in the signalling pathway. Syk has been reported to have a role in signalling in the hematopoietic cells and is implicated in signalling of non-hematopoietic cell types as well [10, 11]. The Syk inhibitor, BAY 61-3606, is reported to have anti-inflammatory effects both *in vitro * and *in vivo * [12]. Na-Bu and other butyric acid derivatives have long been shown to be differentiating and anti-proliferative agents in colonic environment. It is a short chain fatty acid which has differentiation properties in both *in vitro * and *in vivo * conditions [13, 14]. Na-Bu is a specific inhibitor of histone deacetylase (HDAC) [15].

Disrupted kinases are reported in many human cancers and they are implicated in cell cycle and metastasis. Therefore, kinase inhibitors have been developed as cancer therapeutics [16]. In this study, we have analysed the effect of Na-Bu, CDK inhibitor (CDKi), and BAY 61-3606 on the survival of VS tumour cells *in vitro*. A comparative analysis between the untreated control and the treated VS tumour samples was carried out in order to evaluate the effects of these components on the VS tumour cell survival and also to estimate the levels (increase or decrease) of some of the key proteins known to be involved in cell proliferation/death. Exposure of VS cells to these inhibitors caused change in cellular morphology and induced cell death in these cells *in vitro*. We report the modulation of pRb phosphorylation by these inhibitors and their possible role in VS tumour cell survival.

## Materials and methods

### VS tumour tissue and primary culture

This study was approved by the National Institute of Mental Health and Neuro Sciences (NIMHANS, Bangalore, India) human ethics committee which is in accordance with Indian Council of Medical Research (ICMR, India) ethical guidelines for biomedical research on human subjects (2000). Informed consent was obtained from all patients prior to the collection of tissue samples. A total of 16 fresh VS tumour tissues were collected from the NIMHANS Neurosurgery Department during the years 2008–2011. The cell culture media, antibiotic, and the serum were obtained from Sigma-Aldrich, St. Louis, MO. The VS tumour tissues were minced into small pieces and used in this study, and no enzymatic digestions such as collagenase or trypsin which are generally employed to release loose cells from solid tissues were applied to disrupt the tumour tissue or tumour cell integrity. Therefore, the tumour tissues retained all the biological properties such as cell–cell contact, cell integrity, and tumour cell microenvironment as they existed in the patients *in vivo*. The pieces of tumour tissues were placed in DMEM containing 10% fetal bovine serum (FBS), 100 units/ml of penicillin, 100 μg/ml of streptomycin, minced into small pieces, tweezed with forceps and loose cells were released during this process. These were then cultured in Petri dishes (Sigma-Aldrich, St. Louis, MO) and incubated in a humidified atmosphere of 5% CO_2_ and 95% air at 37°C. The untreated control and Na-Bu, CDKi, and BAY 61-3606 treated samples were processed for Western blots and DNA analysis. The time of addition of the three individual components was taken as “zero” hour. Only one VS tumour sample was used for the time course experiment. After estimating the optimum time point (estimated as 48 h), the remaining 16 VS tumour samples were treated for 48 h only and used for DNA fragmentation and Western blot analysis.

### Confirmation of VS tissue

VS tumours were confirmed by routine methods including histopathology, MRI scan, and presence of S-100A2 marker antigen.

### BAY 61-3606, CDKi, and Na-Bu treatments

Na-Bu, CDKi (2,4-diamino-5-oxo-pyrimidine hydrochloride) and BAY 61-3606 (2-[[7-(3,4-dimethoxyphenyl)imidazol[1,2-c]pyrimidin-5-yl] amino]pyridine-3-carboxamide hydrochloride) were obtained from Sigma-Aldrich, St. Louis, MO. The stock solutions were prepared in water. Twenty four hours after seeding the VS tumour tissues to the Petri plates, one set of plates was left untreated and used as control, and the others were treated only once with Na-Bu, CDKi, or BAY 61-3606 at a final concentration of 2 mM [13], 5 nM [17], and 10 nM [12], respectively. Concentrations (1, 2, and 5 mM for Na-Bu; 0.5, 1.0, and 5.0 nM for CDKi; 2, 5, and 10 nM for BAY 61-3606) and time-dependent (24, 48, and 72 h) Caspase-3 and PARP-1 expressions were analysed in order to optimise the concentrations of these inhibitors. VS tumour samples were processed for DNA analysis and western blotting over the time period of 24, 48, and 72 h.

### DNA isolation

Total DNA from the untreated and the treated VS cells were isolated using Tri-reagent (Sigma-Aldrich, St. Louis, MO) according to the protocol supplied by the manufacturer. Ten micrograms of each DNA sample were separated using a 1% agarose gel, stained with ethidium bromide, visualized under UV light, and photographed.

### Western blotting

Qualitative and quantitative analysis of the pRb protein in the untreated and treated tumour samples were done by Western blotting as previously described [18, 19]. The tumour samples were washed thrice in PBS, resuspended in 500 μl of lysis buffer, and lysed using polytron tissue homogenizer (Kinematica AG, Switzerland) clarified by centrifugation, and the supernatant was transferred to a fresh Eppendorff tube. One hundred fifty microgram of each lysate was separated on 7.5% SDS-PAGE gels and transferred to PVDF membrane (Sigma-Aldrich, St. Louis, MO). A 9% SDS-PAGE gel was used to detect the 11-kDa S-100A2 protein. High-molecular weight pre-stained marker (Santa Cruz Biotechnology, Santa Cruz, CA) was used as molecular weight standard. All the gels were run in duplicates, and one set was probed with total pRb antibody or phospho-specific p-Rb antibodies or TGF-β1, JNK1, ASK1, EGF-R, NF-κB, PI3K, Caspase-3, PARP-1, p21, SHH, and S-100A2 antibody. The JNK antibody used is a phospho-specific antibody which will recognise only the 46-kDa pJNK and it did not recognise 54 kDa or any other form of JNK. The 20-kDa SHH protein band is a processed as functional form. The antibody used for the NF-kappa B detects the 65-kDa subunit of endogenous levels of active NF-kappa B protein with pro-inflammatory function. No other isoforms were recognised by this antibody. The other membrane was probed with γ-tubulin antibody (Sigma-Aldrich, St. Louis, MO) which acted as an internal loading control. The pRb antibodies used in this study were as described previously [19]. Anti-β1, JNK, NF-κB, PI3K, p21, SHH, Caspase-3, PARP-1, and S-100A2 antibodies were purchased from Sigma-Aldrich, St. Louis, MO. Anti-EGF-R and ASK1 antibodies were purchased from Oncogene Research Products, Boston, MA. Western blotting was carried out as described previously [19].

The level of total pRb [110 kDa hypophosphorylated (pRb) + 112–114 kDa hyperphosphorylated bands (ppRb)] was measured using Bio-Rad (Bio-Rad, Australia) quantity 1 software for all the samples individually, and these values were normalized to the corresponding control 48 kDa γ-tubulin band from the same samples. The total pRb level for each sample was taken as 100%, and the percentage of phosphorylated form of ppRb in the untreated and the treated samples was estimated by calculating the corresponding (112–114 kDa) band intensities. We have also analysed the expression of TGF-β1, JNK1, ASK1, EGF-R, NF-κB, PI3K, Caspase-3, PARP-1, p21, SHH, and S-100A2 proteins in the lysates at 24, 48, and 72 h, and γ-tubulin was used as loading control in these experiments as well. The results were compared between the untreated and treated VS samples.

### Statistical analysis

Statistical analysis of the data was performed using Statistical Package for Social Sciences (SPSS) version 18.0. Paired *t * test was performed for all the values. Generalized linear model Repeated Measures Analysis of Variants (RMANOVA) one-way classification was used. The overall levels of pRb protein between the untreated and CDKi and BAY 61-3606 treated samples were analysed for statistical significance. For statistical analysis, the percentages of phosphorylated pRb in the untreated and treated samples were transformed to angular transformation. The significance in the levels of phosphorylated form of pRb between the untreated and treated samples was also analysed. The significance in the levels of other proteins involved in the signalling pathways was also analysed between the treated and untreated samples. No statistical analysis was performed for the time course experiments as it was done with one tumour sample. However, statistical analysis was performed for the pooled data obtained from 16 VS tumour samples, 48 h post-treatments.

## Results

### Patient information

The VS tumours were visualized by MRI/CT scans. Histological sections stained with hematoxylin and eosin (H&E) showed characteristic whirling pattern and the presence of Antony A and Antony B regions characteristic of VS tumours (data not shown). Among the 16 patients, 10 were males and 6 were females, and 7 patients belonged to the younger age group of 35 years or below and the remaining 9 were above 35 years of age. All the patients had very large tumours of approximately 3.5–3.8 cm in diameter or larger (Table 1).

### Effects of Na-Bu, CDKi, and BAY61-3606 treatments on morphology and DNA fragmentation in VS cells

**Figure 1:**
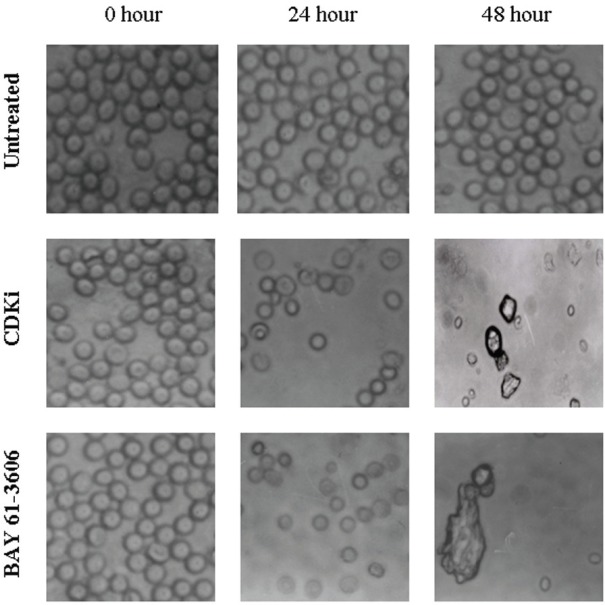
Effect of CDKi and BAY 61-3606 on VS cell morphology. The treatments and the duration are as shown in figure; magnification ×400. The cell morphology data for the Na-Bu treated cells are published previously [19].

**Figure 2:**
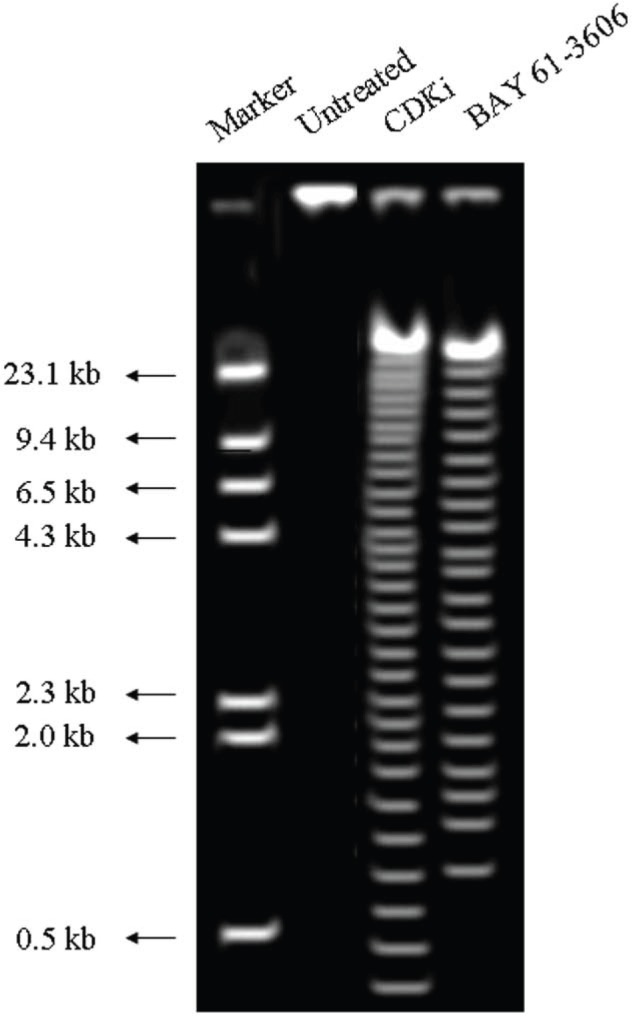
DNA laddering observed in CDKi and BAY 61-3606 treated VS tumor cells at 48 h. The treatments and the molecular weight markers are as indicated in the figure. The DNA laddering data for the Na-Bu treated cells are published previously [19].

We found individual VS cells detached from the fresh VS tumour tissues when they were placed in culture medium and minced with a blade. These cells became loose without any enzymatic digestions or biochemical treatments of the VS tumour tissues. Therefore, physiologically the pieces of tissues used in these experiments *in vitro * had the cell–cell contact and other biochemical properties similar to the tumour tissues as existed within the patients as the tissue integrity was not altered with drastic enzymatic digestions. The primary cultures used in this study therefore contained all the components of the VS tumour in the patient *in vivo*. The loose cells initially appeared round (Figure 1) and took approximately 12–24 h to loosely attach to the cell culture plates. Only after a period of 10–15 days, they attached firmly to the plates and the lamellipodial structures began to appear (data not shown). After 48 h of treatment, more than 90% of the treated cells clumped together as compared to the untreated ones which did not show any morphological change (Figure 1). The total DNA isolated from the treated VS cells showed extensive fragmentation. The extent of fragmentation was higher in the CDKi-treated samples compared to Na-Bu- and BAY 61-3606-treated cells. The total DNA isolated from the untreated control VS samples did not show any fragmentation. Representative data from one sample are shown here (Figure 2). Similar results were obtained in all 16 tumour samples analysed (data not shown).

### Effects of Na-Bu, CDKi, and BAY 61 treatments on pRb phosphorylation

**Figure 3:**
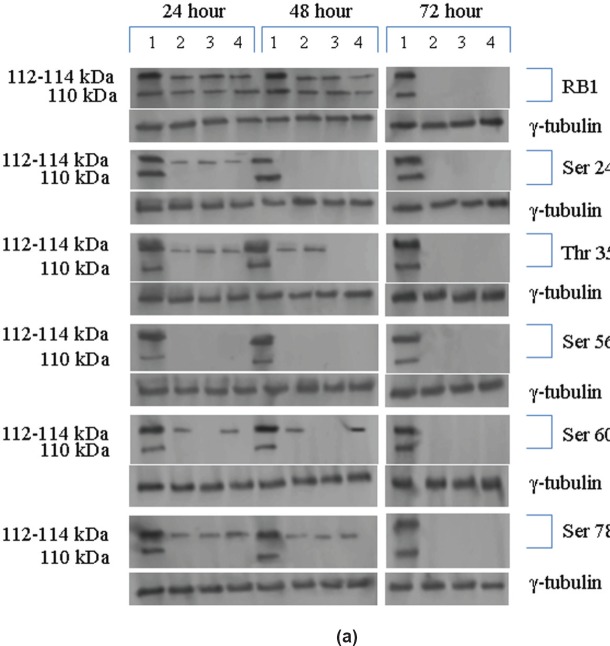

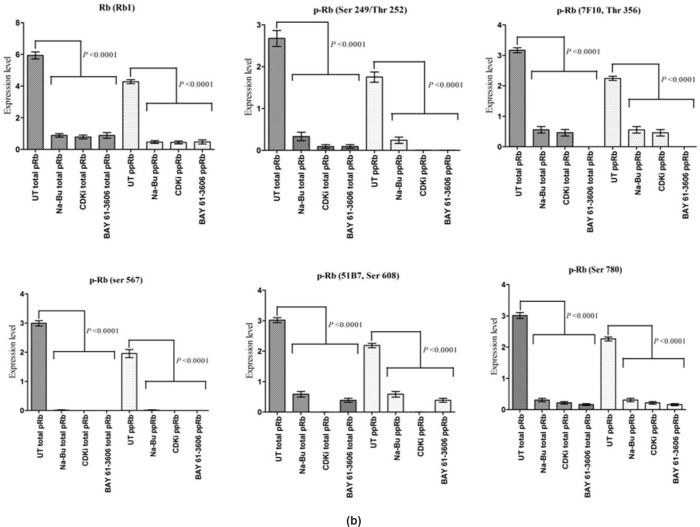

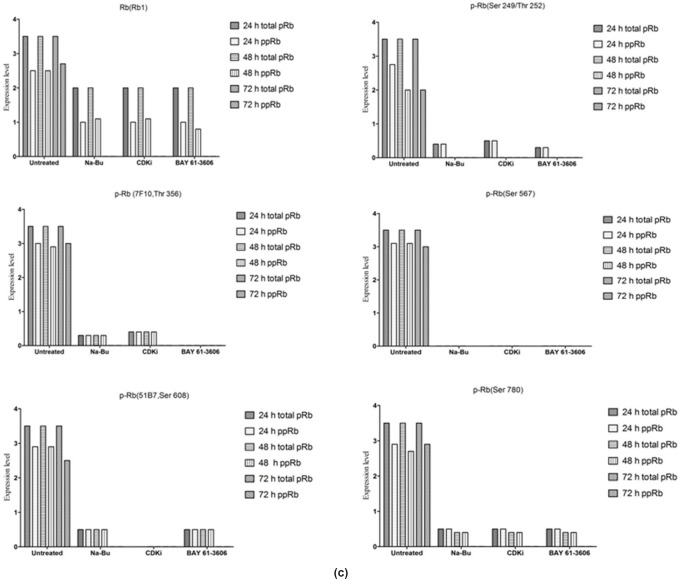
(a) Western blot analysis of total and various site specific phosphorylations in pRb in human VS tumour cells over the time period of 24, 48, and 72 h. 110 kDa hypophosphorylated (pRb) and 112–114 kDa hyperphosphorylated (ppRb) proteins. *Lane 1* untreated; *Lane 2* +Na-Bu; *Lane 3* +CDKi; *Lane 4* +BAY 61-3606. γ-tubulin was used as an internal loading control. (b) Phosphorylation status of pRb for all the 16 tumour samples at 48 h. Error bars are provided for the cumulative expression levels, *n* = 16. Data represented as ±SEM. Statistical tests were done for total pRb and ppRb status between the untreated and treated samples for the six phosphorylation sites analyzed. Effect of treatments on pRb phosphorylation was significant and represented as *P* values. (c) Phosphorylation status of total and various phosphorylation sites of pRb of one representative sample for the time period of 24, 48, and 72 h. No statistical analysis was performed. Antibodies used are as indicated in the figure.

The Rb1 antibody recognized both underphosphorylated and phosphorylated forms of pRb. There was a significant decrease in the overall level of total and phosphorylated forms of pRb in the treated cells as compared to the untreated control samples. Forty eight hours of treatment with Na-Bu, CDKi, and BAY 61-3606 lead to a 60–70% decrease in the levels of total as well as phosphorylated pRb (Figure 3a and b). Only a trace level of total pRb was found at 72 h with all the three individual treatments (Figure 3C). Phosphorylation at the amino terminal Ser 249/Thr 252 site was dramatically reduced with all the three individual treatments at 24 h and was undetectable at 48 and 72 h (Figure 3a–c). Phosphorylation at Thr 356 was reduced by 80% with Na-Bu and CDKi treatments at 24 and 48 h and was undetectable at 72 h after treatment. Thr 356 phosphorylation was completely abolished as early as 24 h with BAY 61-3606 treatment (Figure 3a–c). Ser 567 site was completely de-phosphorylated as early as 24 h post-treatment making it as one of the initial and common responses with all the three treatments (Figure 3a–c). Phosphorylation at Ser 608 was reduced by 70% with Na-Bu and BAY 61-3606 treatments at 24 and 48 h and was undetectable at 72 h. Treatment with CDKi completely abolished phosphorylation at Ser 608 as early as 24 h (Figure 3a–c). The carboxy terminal Ser 780 site was almost equally de-phosphorylated upon the three treatments at 24 and 48 h, and phosphorylated protein was undetectable at 72 h post-treatment (Figure 3a–c). Decrease in the level of total and phosphorylated forms of pRb in the treated samples as compared to the untreated control were statistically significant with a *P* < 0.0001.

### Effects of Na-Bu, CDKi, and BAY 61-3606 treatments on the expression of TGF-β1, JNK1, ASK1, EGF-R, NF-κB, PI3K, Caspase-3, PARP-1, p21, SHH, and S-100A2

**Figure 4:**
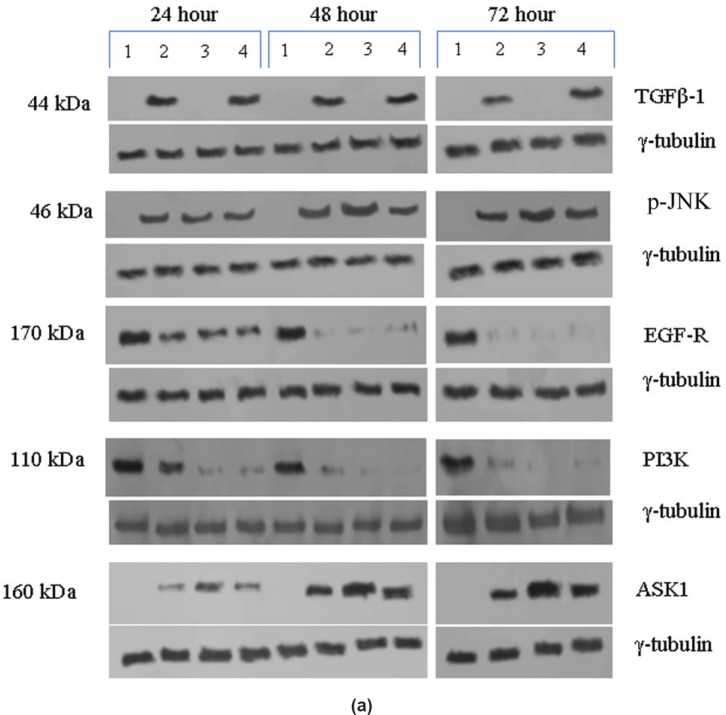

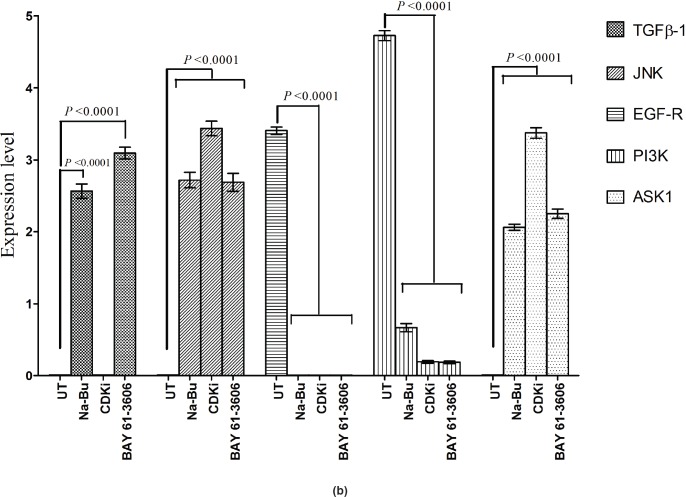

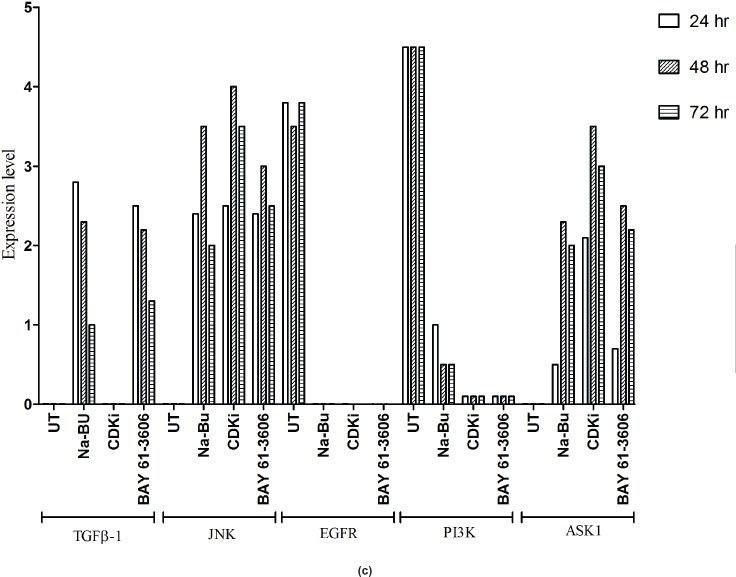
(a) Western blot analysis of TGF-β1, JNK, EGF-R, PI3K, and ASK1 proteins for the time period of 24, 48, and 72 h. *Lane 1* untreated; *Lane 2* +Na-Bu; *Lane 3* +CDKi; *Lane 4* +BAY 61-3606. γ-tubulin was used as an internal loading control. (b) Levels of TGF-β1, JNK, EGF-R, PI3K, and ASK1 proteins 48 h after various treatments. Error bars are provided for the cumulative expression levels, *n* = 16. Data represented as ±SEM. Statistical analysis was done to test the significance of these protein levels between untreated and treated VS tumour samples. Effects of treatments on the level of expression of these proteins were significant and represented as *P* values. Expression of TGF-β1 protein was not significant with CDKi treatment. (c) Expression levels of TGF-β1, JNK, EGF-R, PI3K, and ASK1 proteins for the time period of 24, 48, and 72 h after treatments from one representative tumour sample. No statistical analysis was performed.

In the untreated tumour samples, the TGF-β1, JNK, and ASK1 antibodies did not recognise any detectable level of corresponding proteins by Western blotting. Na-Bu treatment showed a dramatic increase of the 25-, 48-, and 160-kDa TGF-β, JNK, and ASK1 proteins, respectively (Figure 4a–c), and the maximum levels were observed at 48 h. Only the 46-kDa phospho-JNK, which is the predominant form, was recognised by the antibody and the 54-kDa band was not recognised. BAY 61-3606 treatment also resulted in significant increase in the levels of these proteins, and maximum levels were observed at 48 h (Figure 4a–c). CDKi treatment led to induction of only JNK and ASK 1 proteins but not TGFb-1 protein (Figure 4a–c). The results were consistent for all the 16 tumour samples analysed (Figure 4b).

All the untreated samples showed presence of high level of 185 kDa EGF-R protein. However, the level of EGF-R protein was drastically reduced with all three treatments to the extent where it was undetectable by Western blotting. This decrease was observed as early as 24 h post-treatment making it one of the earliest changes in the treated cells. Little change was observed in the EGF-R level in the untreated cells over the time period of 72 h (Figure 4a–c).

**Figure 5:**
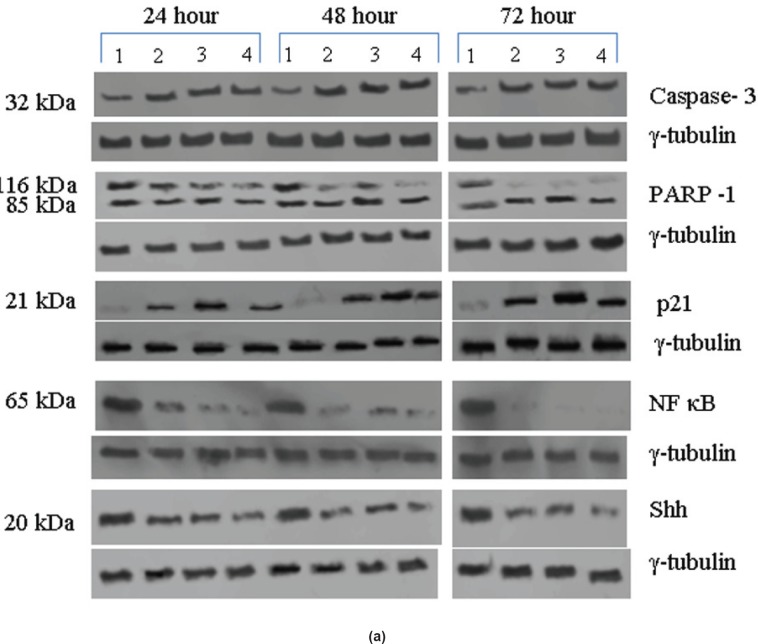

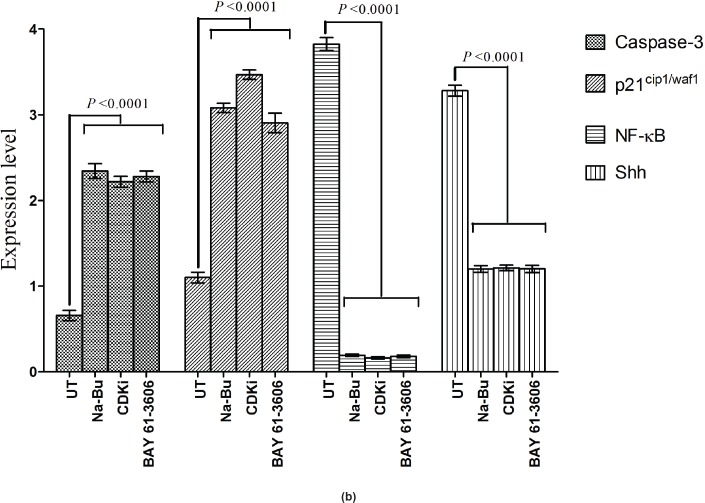

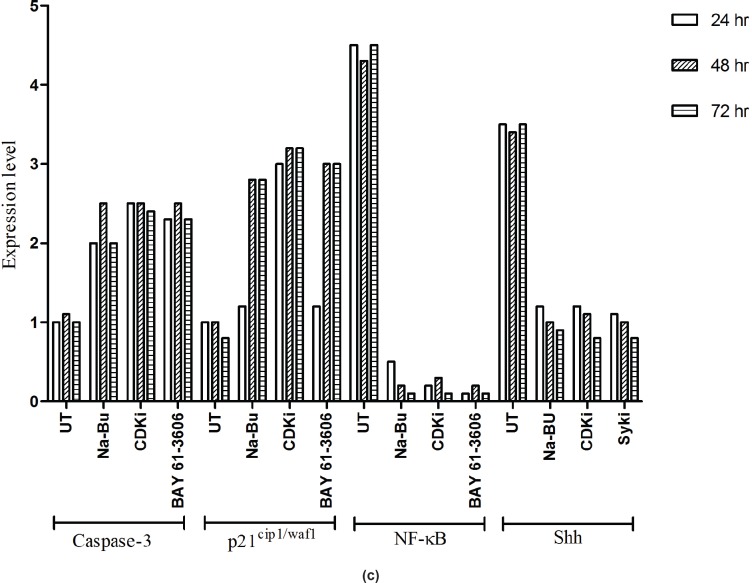
(a) Western blot analysis of Caspase-3, PARP-1, p21, NF-κB, and Shh proteins for the time period of 24, 48, and 72 h. *Lane 1* untreated; *Lane 2* +Na-Bu; *Lane 3* +CDKi; *Lane 4* +BAY 61-3606. γ-tubulin was used as an internal loading control. (b) Expression levels of Caspase-3, p21, NF-κB, and Shh proteins at 48 h. Error bars are provided for the cumulative expression levels, *n* = 16. Data represented as ±SEM. Statistical tests were done in order to test the significance of expression status of different proteins among untreated and treated samples. (c) Expression levels of Caspase-3, p21, NF-κB, and Shh proteins for the time period of 24, 48, and 72 h. No statistical analysis was performed.

The antibodies to PI3K, NF-κB, and Shh recognised high levels of corresponding 110, 65, and 27 kDa proteins in the untreated samples. These levels decreased approximately 90% (PI3K), 95% (NF-κB), and 60% (Shh) after 48 h of treatment as compared to the untreated control (Figure 5a–c). The untreated cells had lower levels of 33 and 21 kDa Caspase 3 and p21^cip1/waf1^ proteins, respectively, and these levels increased by 2.0–3.0-fold after 48 h of all the three individual treatments (Figure 5a–c). No significant changes in the levels of PI3K, NF-κB, Shh, Caspase 3, and p21^cip1/waf1^ proteins were found in the untreated control (Figures 4c and 5c).

Expression of high-levels of 116 kDa PARP-1 and trace levels of 85 kDa cleaved PARP-1 protein was found in the untreated control cells. After treatments, there was an increase in 85 kDa band and a corresponding decrease in the 116-kDa band (Figure 5a). However, the total level of PARP-1 protein remained almost unchanged in the treated and untreated samples (Figure 5a).

**Figure 6:**
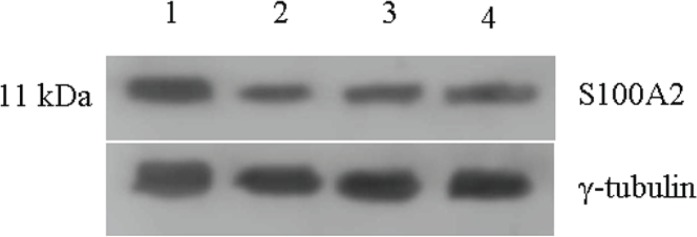
Expression of S-100A2 protein in untreated and 48 h treated VS tumour samples. *Lane 1* untreated; *Lane 2* +Na-Bu; *Lane 3* +CDKi; *Lane 4* +BAY 61-3606. γ-tubulin was used as an internal loading control.

The levels of S100A2 remained higher in untreated control and this level decreased by 70% upon all three individual treatments (Figure 6).

Statistical analysis of the data indicated that the increase or decrease in the levels of the various protein expression patterns in the treated samples as compared to the untreated controls was significant at 48 h post-treatment with a *P * < 0.0001, and this was true for all three individual treatments.

## Discussion

For the first time, we have analysed the effects of three components on the survival of VS tumour tissue samples from 16 patients in primary cultures. Our data indicates that a clear pathway could be involved in VS tumour cell death only in the tumour samples subjected to these treatments. The observed changes in the cellular morphology (Figure 1) [19] and DNA laddering (Figure 2) [19] in the treated cells are indicative of cell death (Figure 2) [19]. Part of the data for the Na-Bu treatment, including DNA fragmentation and change in VS cell morphology between the treated and untreated cells and pRb protein status, is already published from our laboratory [19].

Based on the time course experiment, it was evident that optimum morphological and biochemical changes occurred at 48 h post-treatment. Therefore, this time point was selected for further experiments with 16 VS tumour samples (Figures 3c, 4c, and 5c). However, it is important to note that some of the biochemical changes already appeared only in the treated samples as early as 24 h post-treatment (Figures 3c, 4c, and 5c). This indicates that some of these biochemical changes could directly contribute to the observed cell death. The 48-h time point was selected as the optimum time point, and tumour tissues from 16 patients were treated only for 48 h, and the data from these 16 tissue samples were pooled, subjected to statistical analysis and presented (Figures 3b, 4b, and 5b). The data for 48 h (only) post-treatment from 16 tumour samples was similar to that of the data from 48 h post-treatment from the time course experiment done with a single tumour sample. Statistical analysis was performed for the pooled data from 16 samples, and the error bars are shown in Figures 3b, 4b, and 5b. We also found a slight difference in the effect of the three components, for example, TGF-β1 induction was found only in Na-Bu and BAY 61-3606 treated cells and not in CDKi treated cells (Figure 4a and b). Our data indicate that these three components may have diverse effects on the biochemistry of the VS tumour cells leading to the same end result, that is, cell death.

All the experiments in this study were performed with fresh VS tumour tissues. Molecular changes are known to happen during establishment of permanent cell lines, enzymatic treatments of patients’ tumour tissues and even in primary cultures when they are kept for a prolonged period. Often times these changes continue during serial propagation of these cell lines. The gene expression pattern in the normal cells, including normal Schwann cells, will be quite different than that of the VS tumour cells. It may not be appropriate to compare data from fresh VS tumour tissues obtained from patients to the data from established cell lines or other normal cells. Therefore, we have compared data between the treated and untreated VS samples from each patient that were maintained individually in identical *in vitro * conditions. The pooled data from 16 patients were analysed for statistical significance.

The presence of increased levels of activated JNK (Figure 4a and b) and PARP-1 cleavage (Figure 5a) in the treated cells is indicators of cell death. Presence of high levels of EGF-R and PI3K proteins (Figure 4a and b) in the untreated control are indicators of VS tumour viability and proliferative effect.

The carboxy terminal Ser 780 of the pRb protein has been shown to be efficiently phosphorylated in a cell cycle-dependent manner in T98G glioblastoma cell line [20]. In our study, this site is efficiently phosphorylated in the untreated VS tumour samples (Figure 3a–c). Ser 567 located at the A domain of the pocket region is inefficiently phosphorylated during normal cell cycle [21]. Our data show that pRb is efficiently phosphorylated at this site in all the untreated VS tumour samples (Figure 3a–c) which could inactivate its tumour suppressor function in these tumours. Reduced phosphorylation at Ser 608 and Ser 780 residues has been reported in colon cancer cell lines treated with CYC202 (R-roscovitine) which is a potent CDK2-cyclin E inhibitor and these cells undergo cell cycle arrest [22] and our data on the reduced phosphorylation of pRb at these sites in Na-Bu and BAY 61-3606 treated samples is in agreement with these results (Figure 3a–c).

A recent study showed elevated expression of SYK in primary retinoblastoma tissue and retinoblastoma cell lines and BAY 61-3606 induced cell death in these samples with a concomitant increase in active Caspase-3 [23]. Our data showed increased expression of Caspase-3 and simultaneous increase in PARP-1 cleavage over the time period of 24–72 h with all the three treatments (Figure 5a).

Undetectable or very low level of TGF-β1 protein by western blotting in all the untreated human VS samples observed by us (Figure 4a–c) is in agreement with the previous reports where TGF-β1 and TGF-β2 mRNA were shown to be down-regulated in vestibular schwannoma as compared to the peripheral nerve samples [24], and no TGF-β1 expression was found in a cohort of 34 VS tissues [25]. Na-Bu or BAY 61-3606 treatments induced synthesis of TGF-β1 and JNK and its upstream activator ASK1 (Figure 4a–c) which are known to activate signalling pathways leading to apoptotic cell death [26]. To the contrary, CDKi treatment did not induce synthesis of TGF-β which indicates that the cell death caused by the generalised CDK inhibitor could be independent of TGF-β induction (Figure 4a).

All the untreated VS samples had significantly low level of p21^cip1/waf1 ^ protein (Figure 5a–c) and it correlates to the previous report on loss of its expression in VS tumours [27]. The increased p21^cip1/waf1 ^ level by Na-Bu and BAY 61-3606 treatments can be attributed to induction of TGF-β1 (Figures 4a–c and 5a–c). Our data indicates that the increase in p21 protein level in the CDKi-treated VS cells (Figure 5a) may be independent of TGF-β1 up-regulation (Figure 4a). The observed dephosphorylation or reduced phosphorylation of pRb protein in the treated VS samples (Figure 3a–c) could be due to the induction of activated JNK (Figure 3a–c) which is implicated in stabilizing the kinase inhibitor p21^cip1/waf1 ^ [28]. Induction of p21^cip1/waf1 ^ has been shown to dephosphorylate and deplete cellular pRb [29] and our data on the decreased level of phosphorylated ppRb (Figure 3a–c) along with the induction of activated JNK (Figure 4a–c) and increased level of p21^cip1/waf1^ (Figure 5a–c) are in agreement with these findings [28, 29].

In addition to induction in activated phospho-JNK level, we also found a corresponding increase of ASK1 protein with all the three treatments (Figure 4a–c). ASK1 is known to be an upstream regulator of the JNK pathway [30]. ASK1 is a member of MAP kinase kinase kinase (MAPKKK) family, and it activates both JNK and p38 signalling pathways [31]. From our data, it can therefore be stated that all three treatments induce cell death perhaps via MAP kinase component—ASK1-mediated apoptotic pathway.

All the 16 VS tumour samples used in this study showed the presence of EGF-R protein (Figure 4a–c) and this is in agreement with the previously published studies where EGF-R protein was reported to be present in both unilateral and bilateral VS tumours [32–34]. Based on our data, it can be stated that the EGF-R level is correlated to the large tumour size regardless of the age and gender of the patients (Figure 4a–c; Table 1).

PI3K/AKT pathway has been shown to be activated in human VS tumours [34]. EGF-modulated PI3K activation and invasion of HEI-193 cells *in vitro * have been reported in previous studies [36]. Loss of PI3K expression in addition to drastic down regulation of EGF-R expression by all the three treatments (Figure 4a–c) indicates possible disruption of such cell survival pathway.

The data on the increase in the levels of cell proliferative and cell survival proteins such as EGF-R and PI3K (Figure 4a and b) in the untreated control VS cells are indicative of live VS cells with proliferative potency. A dramatic decrease in the levels of these proteins together with increase in p21 protein (Figure 5a and b) and cleaved PARP-1 proteins (Figure 5a) in the treated cells indicate the activation of a cell death pathway upon treatment.

Loss of negative regulation by TGF-β1 has been shown to be a major reason for up-regulation of pro-inflammatory NF-κB gene in head and neck squamous cell carcinoma [37]. Therefore, decrease in the level of the pro-inflammatory NF-κB in Na-Bu and BAY 61-3606-treated VS tumour samples can be partly explained by the concomitant increase in the level of TGF-β1 (Figures 4a–c and 5a–c). Shh has been identified as a putative target for NF-κB-mediated transcriptional regulation [38], and the decreased level of Shh could be correlated to the decreased in pro inflammatory NF-κB protein levels in the treated samples (Figure 5a–c).

Caspase-3 is required for the fragmentation of DNA [39]. Expression of Caspase-3 increased with all the three treatments with simultaneous increase in PARP-1 cleavage over the time period of 24–72 h (Figure 5a). Presence of a basal level of Caspase-3 and cleaved PARP-1 in the untreated VS tumour samples is indicative of active cell death machinery at a basal level in these tumours.

Na-Bu is a naturally occurring, non-toxic substance. Use of Na-Bu as an anticancer agent has been proposed by many researchers for various tumour types [15]. Bay 61-3606 [12], an anti inflammatory agent, is being evaluated as a cancer therapeutic considering cancer as an inflammatory disease. CDKi and a variety of modified CDKi are also being developed as a potential cancer therapeutic. A modified version of CDKi was evaluated in the clinical trials as a potential cancer therapeutic [17].

It appears that all the large VS tumours (3 cm and above in diameter) perhaps have similar characteristics, regardless of the age and gender of the patients. This is evident as only slight variations in gene expression (data in the present manuscript) were observed between the tumour samples. In this study, we have not used any tumour smaller than 3.5 cm in diameter and we have not used any bilateral NF2 tumour samples. The data presented here are preliminary, and the detailed study of the pathways leading to VS tumour cell death is underway. Detailed analysis of the proteins evaluated in this study is warranted in order to understand the actual mechanisms elicited by these proteins. Our preliminary data point towards the possibility to develop therapeutics to perturb the intracellular signalling to control the growth and progression of VS tumours.

## Conclusion

Data from the present study indicate that the levels of key cell survival proteins such as EGF-R and PI3K as well as phosphorylated pRb were drastically reduced only in the treated VS cells. The treatments also caused increased levels of proteins involved in cell death such as Shh, NF-κB, Caspase 3, and cleavage of PARP in the treated cells as compared to the untreated cells.

**Ethical clearance:** This study was approved by NIMHANS Human Ethics Committee, clearance reference numbers: IEC No. RPA/105/07, dated 26 December, 2007 and No. NIMH/67th IEC/2009, dated 25 September 2009.

**Funding:** This study was supported by financial aid from the Department of Science and Technology (DST), Government of India research project number SR/SO/HS-41/2007. RM is a Senior Research Fellow (SRF) supported by the DST project funds.

**Acknowledgement:** The authors would like to thank Dr. B.A. Chandra Mouli, Dr. S. Sampath, Dr. D.P. Shukla, Dr. S. Dwarkanath, Dr. D.I. Bhat, Dr. N. Rao, Dr. M. Ranjan and others including the residents of the NIMHANS Neuro Surgery Department who provided the tumour tissues used in this study. We thank Dr. D.K. Subbakrishna and Ms. Vasuki Prathyusha for their help in statistical analysis. We thank the Department of Science and Technology (DST), Government of India for providing financial assistance for this study through a research project number SR/SO/HS-41/2007(PI-RG). RM is a Senior Research Fellow (SRF) supported by the DST project funds. The funding agency had no role in the study design, analysis and interpretation of data.

**Contributors:** IDB provided the tumour tissues. RM, IB and RG designed the experiments and RM and RG analysed the data, performed the experiments and wrote the manuscript.

## Figures and Tables

**Table 1.** Patient information.

S. No.	Patient number^a^	Age (years)	Sex	Tumour size (cm)	Symptoms
1	7	55	Male	5.2 × 3.3 × 3.5	Headache
2	9	55	Female	4.4 × 3.2 × 3.1	Speech problem, dysphagia, occasional giddiness, minimal loss of hearing in the right and profound loss of hearing on the left
3	10	60	Female	4.8 × 3.6 × 3.7	Hearing dysfunction, tinnitus
4	11	26	Male	3.5 × 3.1 × 3.3	Headache, profound hearing loss
5	12	32	Female	3.6 × 3.3 × 2.6	Headache, vomiting, hearing loss in left ear
6	13	32	Male	3.5 × 3.5 × 3.2	Tinnitus, deviation of angle of mouth
7	14	40	Male	4.5 × 2.8 × 3.5	Not available
8	15	35	Male	3.9 × 4.3 × 3.6	Not available
9	16	26	Female	4.4 × 3.2 × 3.9	Hydrocephalus
10	17	35	Female	4.2 × 3.2 × 3.7	Swinging while walking, hydrocephalus
11	18	44	Male	3.7 × 3.2 × 3.1	Hearing loss in right ear, tinnitus
12	19	35	Male	3.0 × 3.6 × 2.5	Profound hearing loss in right ear, swinging towards right side
13	20	43	Male	4.2 × 3.7 × 3.8	Headache, vomiting, blurring of vision, tinnitus
14	21	51	Female	3.0 × 2.6 × 2.9	Progressive hearing loss in left ear, difficulty in walking, blurring sensation in left eye
15	22	50	Male	4.0 × 3.9 × 3.8	Swinging while walking, headache, tinnitus, mild hydrocephalus
16	23	26	Male	5.17 × 4.95 × 3.8	Partial deafness, facial palsy, hydrocephalus
After surgery, the tumours were confirmed as VS by histological evaluation of the sections of the tumour tissues					
*Number of males* 10; *number of females* 6; *mean tumour size* 4.1 × 3.5 × 3.3 cm					

a The patient number is assigned in the lab
